# Hypothermia for encephalopathy in low and middle-income countries (HELIX): study protocol for a randomised controlled trial

**DOI:** 10.1186/s13063-017-2165-3

**Published:** 2017-09-18

**Authors:** Sudhin Thayyil, Vania Oliveira, Peter J. Lally, Ravi Swamy, Paul Bassett, Mani Chandrasekaran, Jayashree Mondkar, Sundaram Mangalabharathi, Naveen Benkappa, Arasar Seeralar, Mohammod Shahidullah, Paolo Montaldo, Jethro Herberg, Swati Manerkar, Kumutha Kumaraswami, Chinnathambi Kamalaratnam, Vinayagam Prakash, Rema Chandramohan, Prathik Bandya, Mohammod Abdul Mannan, Ranmali Rodrigo, Mohandas Nair, Siddarth Ramji, Seetha Shankaran, for the HELIX Trial group, Seetha Shankaran, Gaurav Atreja, Mani Chandrasekaran, Jethro Herberg, Pete Lally, Josephine Mendoza, Paolo Montaldo, Vania Oliveira, Ravi Swamy, Sudhin Thayyil, Neil Sebire, Nigel Klein, Kshitij Mankad, Arjun Chandra Dey, Sanjoy Kumer Dey, Mohammed Tariqul Islam, Ismat Jahan, Mohammed Abdul Mannan, Sadeka Chowdhury Moni, Kamrul Hasan Shabuj, Mohammod Shahidullah, Mohammed Nazrul Islam, Mst. Nazmun Nahar, Ashish Jain, Siddarth Ramji, Swati Manerkar, Jayashree Mondkar, Kapil Dewang, Swapnil Bhiskar, Chinnathambi Kamalaratnam, Kumutha Kumaraswami, Sundaram Mangalabharathi, Padmesh Vadekepad, Monica Sebastian, Naveen Benkappa, Prathik Bandya, Usha Kantharajanna, Sowmya Krishnappa, Jagdish Somanna, Niranjan Hunsanhalli Shivanna, Arasar Seeralar, Vinayagam Prakash, Mythilli Babu, Mohamed Sajjid, Babu Peter Sathyanathan, Kailasanathan Natarajan, Senthil Kumaran, Anusha Rohit, Indrani Karunasagar, Vijaykumar Madhavan, Mohandas Nair, Ranmali Rodrigo, Shaman Rajindrajith, Jithangi Wanigasinghe, Samanmali Sumanasena, Radika Karunaratne, Sanjeewa Munasinghe, Kalpani Chathirangika

**Affiliations:** 1 Centre for Perinatal Neuroscience, Imperial College London, London, UK; 2 Stats Consultancy, Amersham, Buckinghamshire UK; 3 Lokmanya Tilak Municipal Medical College, Sion, Mumbai, India; 4 Institute of Child Health, Egmore, Madras Medical College, Chennai, India; 5 Indira Gandhi Institute of Child health, Bangalore, India; 6 Institute of Obstetrics & Gynecology, Madras Medical College, Chennai, India; 7 Neonatal Medicine, Bangabandhu Sheikh Mujib Medical University, Dhaka, Bangladesh; 8 Paediatric Infectious Diseases, Imperial College London, London, UK; 9 University of Kelaniya, Kelaniya, Sri Lanka; 10 Institute of Maternal and Child Health, Government Medical College Calicut, Calicut, India; 11 Maulana Azad Medical College, New Delhi, India; 12 Neonatal-Perinatal Medicine, Wayne State University, Detroit, MI USA

## Abstract

**Background:**

Therapeutic hypothermia reduces death and disability after moderate or severe neonatal encephalopathy in high-income countries and is used as standard therapy in these settings. However, the safety and efficacy of cooling therapy in low- and middle-income countries (LMICs), where 99% of the disease burden occurs, remains unclear. We will examine whether whole body cooling reduces death or neurodisability at 18–22 months after neonatal encephalopathy, in LMICs.

**Methods:**

We will randomly allocate 408 term or near-term babies (aged ≤ 6 h) with moderate or severe neonatal encephalopathy admitted to public sector neonatal units in LMIC countries (India, Bangladesh or Sri Lanka), to either usual care alone or whole-body cooling with usual care. Babies allocated to the cooling arm will have core body temperature maintained at 33.5 °C using a servo-controlled cooling device for 72 h, followed by re-warming at 0.5 °C per hour. All babies will have detailed infection screening at the time of recruitment and 3 Telsa cerebral magnetic resonance imaging and spectroscopy at 1–2 weeks after birth. Our primary endpoint is death or moderate or severe disability at the age of 18 months.

**Discussion:**

Upon completion, HELIX will be the largest cooling trial in neonatal encephalopathy and will provide a definitive answer regarding the safety and efficacy of cooling therapy for neonatal encephalopathy in LMICs. The trial will also provide important data about the influence of co-existent perinatal infection on the efficacy of hypothermic neuroprotection.

**Trial registration:**

ClinicalTrials.gov, NCT02387385. Registered on 27 February 2015.

**Electronic supplementary material:**

The online version of this article (doi:10.1186/s13063-017-2165-3) contains supplementary material, which is available to authorized users.

## Background

Every year, approximately one million babies die in low- and middle-income countries (LMIC) due to neonatal encephalopathy – a condition arising from an unexpected lack of cerebral blood flow and oxygen supply to the fetal brain at the time of birth [1]. Approximately one-third of infants with moderate or severe encephalopathy will die during the newborn period and up to three-quarters of survivors will develop long-term neurodisability [2, 3]. Until recently, there was no effective treatment for this condition and the management was limited to supportive care.

Several high-quality cooling trials have been conducted in high-income countries in the past decade [4–6]. The meta-analyses of these trials have convincingly demonstrated that selective head or whole-body cooling, along with optimal tertiary intensive care, reduces mortality (risk ratio [RR] 0.8; 95% confidence interval [CI] 0.7–0.9; *p* = 0.005) and improves survival with normal neurological outcome (RR 1.5; 95% CI 1.2–1.9; *p* < 0.001) after neonatal encephalopathy in these settings [7, 8]. The protective effect of cooling persists into later childhood [9, 10]. Whole-body cooling is now widely used as a standard therapy for encephalopathy in the UK and other high-income countries [11].

The safety and efficacy data on cooling therapy from high-income cooling trials cannot be extrapolated to LMICs, due to differences in population co-morbidities, particularly co-existent perinatal infection, growth restriction and meconium aspiration syndrome, and lack advanced cardio-respiratory intensive care facilities [12]. Preclinical data suggest that the brain injury is worse and the neuroprotective effect of hypothermia in neonatal encephalopathy is lost in the presence of co-existent infection, particularly with gram-negative organisms [13].

We systematically reviewed the published literature on the safety and efficacy of cooling therapy for neonatal encephalopathy due to hypoxia-ischemia in LMICs [14]. All published studies were small and/or of poor quality. Two studies reported increased mortality with cooling [15, 16]. A meta-analysis of all these trials showed a trend towards reduced mortality; however, this was not statistically significant (RR 0.74; 95% CI 0.4–1.3). More importantly, the CIs were wide and therefore significant benefits or harm could not be excluded. There were no data on long-term neurological follow-up after cooling therapy [14]. Thus, before cooling therapy is widely used as standard care therapy in LMICs, safety and efficacy data from adequately powered clinical trials are required.

### Aims

#### Primary

To examine whether whole-body cooling to 33.5 °C, initiated within 6 h of birth and continued for 72 h, reduces death or moderate or severe neurodisability at 18–22 months after neonatal encephalopathy, in LMICs.

#### Secondary

- To examine if whole-body cooling reduces mortality at hospital discharge and at 18–22 months after neonatal encephalopathy
- To examine if whole-body cooling reduces moderate or severe neurodisability at 18–22 months in survivors after neonatal encephalopathy
- To examine if whole-body cooling reduces brain injury on magnetic resonance imaging (MRI) and spectroscopy performed during the neonatal period

## Methods

This is a multi-country two arm unblinded pragmatic randomised controlled trial of whole-body cooling along with usual supportive care vs. usual care alone. We plan to recruit 408 babies with moderate or severe neonatal encephalopathy from public sector neonatal units in LMICs over a three-year period. We anticipate approximately 1200 babies will be screened for eligibility to achieve the required target.

The treatment duration (cooling therapy) is 72 h; however, the temperature of all recruited babies will be monitored during the first week after birth. Any temperature rise > 37.5 °C will be actively treated, both in the cooling and usual care arms, as fever increases brain injury and adverse outcomes after neonatal encephalopathy. The neurological outcomes will be assessed at 18–22 months of age. The trial duration will be five years, consisting of a staggered start-up period, 36 months of recruitment followed by a further 18 months of follow-up, and a final five months for data analysis and write-up.

Before the start of recruitment, the local clinical staff at the recruiting centres will have an intensive training on all aspects of the trial and research governance. All staff involved in the study will be required to maintain an up-to-date ICH-GCP certification.

### Inclusion criteria

To ensure wider applicability of the trial intervention, we will use exclusive clinical criteria for selection of at risk infants. Although blood gas analysis and amplitude integrated electroencephalogram may be performed as a part of the clinical care, they will not form part of the eligibility criteria, as these investigations are not widely available in LMICs. Furthermore, accurate Apgar scores are often not available when babies are born at home or in other hospitals and then transferred to the recruiting centres, and often not collected beyond 5 min after birth. Hence, the inclusion criteria from the high-income country cooling trials are inappropriate for LMICs.

Therefore, our inclusion criteria are primarily based on a structured neurological examination using modified Sarnat staging.

Our preliminary data have shown that the HELIX inclusion criteria will identify most infants at high risk of adverse outcomes, without including infants with milder encephalopathy. To be eligible for recruitment, all three criteria below must be met:

1. age ≤ 6 h, birthweight ≥ 1.8 kg, gestation ≥ 36 weeks (based on reported last menstrual period or ultrasound);
2. need for continued resuscitation at 5 min of age and/or 5-min Apgar score < 6 (for babies born at hospital) or lack of crying by 5 min of age (for babies born at home);
3. Evidence of moderate or severe encephalopathy at < 6 h of age on a structured clinical examination based on modified Sarnat staging (Table 1).

**Table 1:** Modified Sarnat scale for clinical encephalopathy staging

Categories (total 6)	Signs of neonatal encephalopathy (NE) in each category			
	Normal	Mild NE	Moderate NE	Severe NE
1. Level of consciousness				
	Alert, responsive to external stimuli (state dependent, e.g. post feeds)	Hyper-alert, has a stare, jitteriness, high-pitched cry, exaggerated responds to minimal stimuli, inconsolable	Lethargic	Stupor/coma
2. Spontaneous activity				
	Changes position when awake	Normal or decreased	Decreased activity	No activity
3. Posture				
	Predominantly flexed when quiet	Mild flexion of distal joints (fingers, wrist usually)	Moderate flexion of distal joint, complete extension	Decerebrate
4. Tone				
	Strong flexor tone in all extremities + strong flexor hip tone	Normal or slightly increased peripheral tone	Hypotonia (focal or general) or hypertonia	Flaccid Rigid
5. Primitive reflexes (circle only the highest level in each sign; the maximum score is only 1 in any one category)				
Suck	Strong, easily illicit	Weak, poor	Weak but has a bite	Absent
Moro	Complete	Partial response, low threshold to illicit	Incomplete	Absent
6. Autonomic system (circle only the highest level in each sign; the maximum score is only 1 in any one category)				
Pupils	In dark: 2.5–4.5 mm; in light: 1.5–2.5 mm	Mydriasis	Constricted	Deviation/dilated/non-reactive to light
Heart rate	100–160 bpm	Tachycardia (HR > 160)	Bradycardia (HR < 100)	Variable HR
Respiration	Regular respirations	Hyperventilation (RR > 60/min)	Periodic breathing	Apnoea or requires ventilator
Total score				

The level of encephalopathy will be assigned based on which level of signs (moderate or severe) predominates among the six categories. If moderate and severe signs are equally distributed, the designation is then based on the highest level in Category #1: The level of consciousness. If the level of consciousness is equal, then designation of the NE stage is based on the tone (Category #4). An infant who has seizures will be moderate or severe NE, depending on the neurologic exam. Seizure with normal or mild NE or moderate NE on neurologic exam will be ‘Moderate NE’. Seizure with severe NE will be ‘Severe NE’

### Exclusion criteria

- Absent heart rate at 10 min of age despite adequate resuscitation or imminent death
- Major life-threatening congenital malformations
- Migrant family or parents unable/unlikely to come back for follow-up at 18 months (expected to be less than 5% of eligible population)

### Outcome measures

#### Primary outcome measure

Severe disability was defined as any of the following: a Bayley III [17] cognitive score < 70; a Gross Motor Function Classification System (GMFCS) level of 3–5 [18]; blindness; or profound hearing loss (inability to understand commands despite amplification).

Moderate disability was defined as a Bayley III cognitive score of 70–84 and either a GMFCS level of 2, seizure disorder or a hearing deficit requiring amplification to understand commands.

#### Secondary outcome measures

##### Clinical outcomes (before discharge from hospital)

- Mortality from any cause before discharge from hospital
- Major intracranial haemorrhage on cranial ultrasound
- Gastric bleeds (fresh blood > 5 mL from nasogastric tube)
- Persistent hypotension (mean blood pressure < 25 mmHg despite maximum inotropic support)
- Pulmonary haemorrhage (copious bloody secretions with clinical deterioration requiring change(s) in ventilatory management)
- Persistent pulmonary hypertension (severe hypoxemia disproportionate to the severity of lung disease with a significant pre- and post-ductal saturation difference on pulse oximetry)
- Prolonged blood coagulation requiring blood products
- Culture-proven early onset sepsis (isolation/identification of a pathogenic organism from blood and/or cerebrospinal fluid along with clinical evidence of sepsis and elevation of C-reactive protein)
- Necrotising enterocolitis (abdominal distension, increased gastric aspirates and/or blood in stools, together with abdominal X-ray showing bowel oedema, pneumatosis or pneumoperitoneum, i.e. Bell’s staging 2 or 3)
- Cardiac arrhythmia requiring therapy
- Severe thrombocytopenia (<25,000)
- Persistent metabolic acidosis lasting over 12 h after birth
- Renal failure (anuria > 48 h with azotaemia)
- Pneumonia (infiltrates on chest X-ray consistent with infection or aspiration)
- Subcutaneous fat necrosis
- Neurological examination at discharge
- Duration of hospitalisation

##### Neonatal cerebral magnetic resonance biomarkers

- Brain injury score on conventional MRI [19, 20]
- Proton magnetic resonance spectroscopy thalamic lactate/N-acetylaspartate peak area ratio and absolute concentration of N-acetylaspartate [21]
- Whole brain maps of diffusion tensor indices [22]

##### Neurodisability (18–22 months)

- Mortality
- Severe neurodevelopmental disability (severe disability was defined as any of the following: a Bayley III [17] cognitive score of < 70; a GMFCS level of 3–5 [18]; blindness; or profound hearing loss (inability to understand commands despite amplification)
- Microcephaly (head circumference more than 2 standard deviations below the mean)

### Screening and neurological examination

All infants admitted to the neonatal unit with perinatal asphyxia will be screened for eligibility. Out-born babies meeting the inclusion criteria will be eligible for recruitment, irrespective of the temperature at admission to the neonatal unit (Additional file 1). Potentially eligible cases will have a detailed neurological examination by a designated neonatal doctor who is trained and accredited in NICHD neurological examination modified from the Sarnat staging (Table 1) [5]. Briefly, this scoring system assesses six categories of neurological symptoms. The highest score in each category will be recorded. The level of encephalopathy is determined based on the predominant degree of neurological abnormality (moderate or severe) across the six categories. Three out of the total six categories must be moderate or severe for the baby to be eligible.

Infants who have seizures will be considered to have moderate or severe neonatal encephalopathy depending on the neurological examination. Seizures combined with otherwise mild or moderate encephalopathy on neurological examination will be considered as moderate encephalopathy. Seizures combined with severe encephalopathy will be considered as severe encephalopathy. Sedatives or analgesics given before the neurological examination will be recorded in the case report form (CRF).

### Treatment assignment and randomisation

As soon as informed parental consent is obtained for an eligible infant, the recruiting clinician will obtain the treatment assignment, which will be either ‘usual care with cooling’ or ‘usual care only’, using an Internet-based randomisation system (Sealed Envelope; https://www.sealedenvelope.com). Minimisation will be used to ensure balance between the groups with respect to the severity of encephalopathy at each centre. Further details are given in the statistical analysis plan.

Researchers will not be blinded to the intervention (cooling therapy). However, the neurological outcome evaluation at 18 months will be undertaken by assessors masked to the treatment allocation.

### Cooling therapy

Therapeutic hypothermia will be administered using a servo-controlled whole-body cooling device, that has an effective cooling time (percentage of time for which the core body temperature is maintained within the target range of 33 °C to 34 °C) of over 90% [23]. Briefly, this would consist of attaching the mattress to the servo-controlled device, refilling coolant, keeping the baby on the mattress, placing a rectal probe, switching the machine on and selecting the appropriate program. Babies are kept on a radiant warmer with heating turned off and are not clothed, except for nappies. The cooling device will maintain the rectal temperature of the baby within 33 °C to 34 °C and will alarm when temperatures are out of this range (e.g. after displacement of the rectal probe). The clinical team will record rectal temperature hourly in the data collection form. In addition, the temperature data will be downloaded from the cooling device and compared with the manual records to ensure data entry accuracy. After 72 h of cooling, the baby will be automatically re-warmed at 0.5 °C per hour by the cooling device. Following re-warming all babies will have continuous rectal temperature monitoring until 90 h of age, and then every 8 h for the first week after birth.

#### Babies with a low temperature (rectal temperature < 35.5 °C) on admission to the neonatal unit

It is possible that rapid rewarming (>0.5 °C per hour) may result in increased brain injury and seizures. If these babies are already randomised to usual care and then kept under a servo-controlled radiant warmer set at 36.5 °C, rapid re-warming may occur in less than 1 h, thus worsening the outcome.

Hence, any baby admitted with a low rectal temperature (<35.5 °C) who is recruited to the usual care arm will be slowly rewarmed, at a rate not more than 0.5 °C per hour using the protocol below:

1. set the radiant warmer temperature 0.5 °C higher than the baby’s rectal temperature and increase this by 0.5 °C every hour, until the rectal temperature reaches 36.5 °C;
2. if the radiant warmer servo-controlled mode fails, use manual mode keeping the heating output low (e.g. 20%), so that the baby’s rectal temperature does not rise by more than 0.5 °C per hour;
3. if no radiant warmer available, keep the baby covered in warm clothes, so that the temperature slowly increases to 36.5 °C over several hours. This may take as long as 6–12 h.

#### Babies with a core temperature of > 37.5 °C during the first week after birth

All the major neonatal cooling trials to date had up to one-quarter of infants in the usual care having core temperatures > 37.5 °C. Subsequently, analysis of the trial data suggests that core temperatures above 37.5 °C adversely affect brain injury and worsen their outcome [24]. Therefore, any potential benefit seen with cooling may be spuriously related to the overheating of usual care babies. Moreover, a recent trial in adults have shown that ‘therapeutic normothermia’ is as effective as ‘therapeutic hypothermia’ following cardiac arrest [25].

Hence, we will use a more aggressive approach for preventing and treating elevated core temperatures in the usual care arm than the previous neonatal cooling trials. Fever may occur at any time in babies in the usual care arm and sometimes during the post-rewarming phase in the cooling arm. Babies allocated to usual care will be nursed on servo-control radiant warmer with a set temperature of 35.5 °C to 36 °C, to prevent any accidental overheating. Any rise in temperature > 37.5 °C, will be aggressively treated by turning off any radiant heater or warmer if in use, using fans, tepid sponging, antipyretics and, if necessary, using the servo-controlled cooling device in a ‘normothermia mode’. In addition, any infective cause for fever will be investigated and treated, including lumbar puncture if appropriate.

### Criteria for stopping cooling therapy

- Refractory hypotension (mean blood pressure < 25 mmHg) despite optimal inotropic and volume support
- Life-threatening/massive haemorrhage
- Parent or clinician request to stop cooling therapy, for example if the baby requires any surgical procedure during first three days

### Supportive care and monitoring

The general management of babies will be standardised at the participating centres and will not permit therapies like steroids, mannitol or other experimental therapies in recruited infants.

Hourly vital signs (oxygen saturation, heart rate, non-invasive blood pressure, rectal temperature) will be recorded in the HELIX CRF in all infants. Additional monitoring will be dictated by the clinical condition and local guidelines.

Infants may also receive intravenous fluids, antibiotics, ventilatory support, inotropes, blood products, sedation, muscle relaxants and anti-convulsants as per the local clinical practice. The babies undergoing cooling therapy will also receive sedation, if they are ventilated or if there is any evidence of stress (for example, shivering, unexplained tachycardia).

Detailed neurological examination (using NICHD criteria) will be performed within 6 h of birth, then at day 3 and at discharge from hospital.

### Baseline assessments and data collection

The following data points will be recorded in the CRF:

1. maternal (antenatal) and delivery details including resuscitation details;
2. time and date of birth, time of randomisation and start of cooling;
3. birthweight, gestation, gender and head circumference;
4. hourly rectal temperature profile in all infants for the first 90 h;
5. neurological examination within 6 h, at day 3 and at the time of discharge;
6. full blood count (including platelets, CRP and differential white cell count) within 6 h of birth and between days 4 and 7, as a part of clinical care;
7. Blood culture within 6 h of birth and between days 4 and 7;
8. biochemical series (including blood gas, sugar, urea, creatinine, electrolytes and coagulation profile) as a part of clinical care;
9. cranial ultrasound examination (within 72 h) to examine for major intracranial bleeds as a part of clinical care.

Each anonymised CRF will be scanned and emailed to the HELIX trial manager at Imperial College London for quality checks within 48 h of completion. The signed off CRFs will be entered into the HELIX trial database (Redcap®).

#### Screening for perinatal infection

**Fig. 1:**
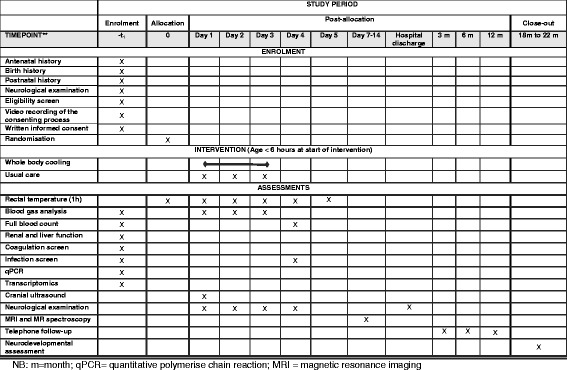
Schedule of enrolment, intervention, and assessments

Preclinical evidence suggests that co-existent bacterial infections, particularly those due to gram-negative bacteria, may negate the neuroprotective effects of hypothermia. Therefore, we will perform advanced molecular, histological and transcriptomic evaluation in addition to standard automated blood cultures (Bactec), to identify any co-existent perinatal infection (Fig. 1). Whenever possible, bloods will be collected before giving antibiotics. Babies who had antibiotics before the blood collection (for example, babies referred from other hospitals) will be noted separately.

This will include:

- blood for targeted polymerase chain reaction (qPCR) to transcriptomic signatures to detect common bacterial pathogens within 6 h of birth;
- a small section of the umbilical cord (fetal end) for histopathological examination for funisitis. In addition, a section from the placenta will be collected whenever feasible. An experienced perinatal pathologist will report histopathology, masked to the clinical data.

We will use multiplex real time PCR (qPCR) on blood to identify babies with neonatal sepsis, using a panel of primers for both specific detection of common pathogens and generic detection. This will include *Enterobacteriacae*, *Fusobacteria* spps., *Staphylococcus aureus*, coagulase-negative *Staphylococci*, and *Streptococcus* spps including Group A and B *Streptococcus*, *Pneumococcus* and *Peptostreptococcus* spps), qPCR for quantitation of total 16 s rDNA [26]. Our preliminary data suggest the vast majority of pathogens are gram-negative, and unlike in high-income countries, group B streptococci is extremely rare in these settings.

Following RNA extraction from the whole blood collected in an RNA stabilising solution, we will perform next generation sequencing (paired end) and alignment. The sequenced data will be examined for the established signatures of bacterial infections recently reported from Imperial College London [27].

#### Magnetic resonance imaging and spectroscopy

The treatment effect of cooling therapy on brain injury will be assessed and quantified using MRI and spectroscopy. All recruited infants (surviving beyond 1 week) will have an MR scan at 7–14 days of age. Preparation of the baby for MRI is given in Additional file 2.

All MR scans will be performed on 3 T scanners (Philips, Siemens or GE) using common harmonised sequences developed as a part of the Magnetic Resonance Biomarkers in Neonatal Encephalopathy (MARBLE) study﻿﻿ [28]. 3 T MR scanning offers considerable benefits for MR spectroscopy, over 1.5 T imaging.

MR scanners will be calibrated using adult volunteer brain scans using the same sequences.

#### Conventional MRI protocol

- T1-weighted – 3D MPRAGE (1-mm isotropic resolution);
- T2-weighted – 2D axial TSE (0.5-mm in-plane resolution, 3-mm slices);
- Diffusion – 2D axial SE-EPI (2-mm isotropic resolution, b = 0.750 s mm^-2^).

#### MR spectroscopy protocol

- Surveys pre- and post-spectroscopy for detection of gross motion;
- PRESS (15 × 15 × 15 mm^3^, water suppressed, echo time = 288 ms) for metabolite peak area ratios;
- STEAM (15 × 15 × 15 mm^3^, water suppressed, dual repetition time, echo time = 20 ms) for relaxation corrected metabolite signals;
- STEAM (15 × 15 × 15 mm^3^, water unsuppressed, fixed repetition time, multiple echo times) for internal water referencing to provide metabolite concentrations.

The anonymised MR data will be encrypted and transferred to the Centre for Perinatal Neuroscience by Imperial College London file transfer protocols for analysis and storage. All MR data will be analysed centrally, masked to the allocation or other outcomes. Details of MR sequences are given in Additional file 3.

The raw spectroscopy data will be post-processed using software developed in-house at Imperial College London (Python v2.7) and an in-house implementation of TARQUIN will be used to calculate metabolite concentrations from the STEAM spectra. LCModel will be used to calculate metabolite peak area ratios from the PRESS spectra.

Diffusion imaging data will be processed using the FMRIB Software Library (FSL, v5.0) and registered across individuals using DTI-TK (http://dti-tk.sourceforge.net/pmwiki/pmwiki.php). The resulting maps of diffusion tensor indices will be compared between participants using FSL. Postnatal age and postmenstrual age at scan will be introduced as confounding variables into the general linear model rendering results age-independent.

#### MR biomarker endpoints

- thalamic N-acetylaspartate concentration determined from STEAM spectroscopy;
- thalamic lactate/N-acetylaspartate metabolite peak area ratio determined from PRESS spectroscopy;
- white matter fractional anisotropy determined from diffusion imaging using tract based spatial statistics;
- brain injury severity score determined from conventional MRI [20].

### Follow-up and neurological assessments

The recruiting centres will maintain regular (3–6 months) telephone contact with parents to minimise attrition. The following information will be recorded at each contact:

1. general health status of the baby;
2. any change in home address or telephone number.

Each recruiting centre will have a dedicated and experienced neurodevelopmental paediatrician trained in Bayley Scales of Infant Development (Version III), who will assess the babies aged 18–22 months, masked to the allocation. Inter-observer variability will be addressed and corrected before the start of assessments, by comparing against a gold standard examiner (RS; Pearson’s trainer for Bayley Scales of Infant Development (Version III). The Bayley scales will be administered in one of the local languages (mother tongue of the child) – Hindi, Marathi, Kannada, Tamil, Malayalam, Singhalese, Telugu or Bangla.

Detailed medical history, neurological examination and Gross Motor Function Classification System assessment will be also obtained during this visit using a pre-defined proforma and then entered into the HELIX trial database.

Severe disability will be defined as any one of the following: Bayley III cognitive composite score < 70; Gross Motor Function Classification System level 3–5; hearing impairment requiring hearing aids/cochlear implant; or blindness.

Moderate disability will be defined as cognitive composite score 70–84 and one or more of the following: Gross Motor Function Classification System level II; hearing impairment with no amplification/cochlear implant; or a persistent seizure disorder.

The examiner will feed back the results of the neurodevelopmental outcome tests to the parents, immediately after the assessment. A copy of this report will be provided to the local principal investigator for clinical management. We will also collect information of various morbidities and medical support required during infancy and obtain detailed anthropometry to assess the nutritional status during the 18-month follow-up visit.

### Adverse events

All known adverse events relating to neonatal encephalopathy and cooling therapy are described in the parent information leaflet and will be part of obtaining the informed research consent, before the start of cooling therapy.

The following clinical events (1–9) occur due to the underlying disease (neonatal encephalopathy). Cooling trials from high-income countries have shown that cooling therapy reduces/does not increase the incidence of many of these clinical events in encephalopathic babies.

1. Death during neonatal period or during infancy
2. Brain injury observed with MRI
3. Adverse neurodevelopmental outcome at 18 months and at childhood
4. Persistent pulmonary hypertension
5. Metabolic imbalances
6. Cardiac arrhythmia
7. Renal failure
8. Coagulopathy
9. Gastric bleeds

Cooling therapy may increase the risk of the following adverse events noted in the previous randomised controlled trials.

1. Thrombocytopenia and increased need for platelet transfusions
2. Subcutaneous fat necrosis

All adverse events are expected to occur within the cooling period (first 72 h) or within 72 h of re-warming. Adverse reactions occurring subsequently (after one week of life), except subcutaneous fat necrosis, will not be considered as intervention related. Subcutaneous fat necrosis may occur several weeks after the therapy.

If an unexpected serious adverse event (SAE) occurs (i.e. an event not mentioned in the above list), it should be reported to the HELIX trial manager within 24 h, using one of the SAE report forms. The HELIX trial manager will ensure that the independent data monitoring committee (IDMC) and the research ethics committee (REC) are informed accordingly.

### Statistical methods

The primary analysis will be a comparison of the infants assigned to usual care plus whole-body cooling with those infants assigned to usual care at randomisation (i.e. intention-to-treat analysis population), regardless of deviation from the protocol or whether they received the allocated intervention. Demographic factors, clinical characteristics and outcomes will be summarised with counts (percentages) for categorical variables, means (standard deviation [SD]) for normally distributed continuous variables or medians (interquartile [IQR] or entire range) for non-normally continuous variables. Further details are given in the statistical analysis plan.

In order to establish both the magnitude and direction of the effects of whole-body cooling intervention, comparative statistical analysis will entail calculating the RR plus 95% CI for the primary outcome. The chi-square test will be used to determine statistical significance, with a 5% significance level used.

Secondary outcomes will be evaluated using a 5% level of statistical significance, with 95% CIs reported, to take account of the number of outcomes analysed. The Chi-square test or Fisher’s exact test will be used to analyse categorical outcomes with RRs reported with 95% CIs. The unpaired t-test will be used to analyse normally distributed continuous outcomes, with the mean difference (plus 95% CI) reported. Non-normally distributed continuous outcomes will be transformed to normality or alternatively analysed using the Mann–Whitney test. If the latter approach is used, the median difference (plus 95% CI) between groups will be reported.

Logistic regression will be used to perform an adjusted analysis for the primary outcome to investigate the impact of stratification/known prognostic factors including the stage of neonatal encephalopathy.

Analysis of secondary outcomes will be clearly delineated from the primary analysis in any statistical reports produced. Results will be reported according to the CONSORT statement.

The sample size is based on being able to detect a clinically significant 30% relative risk reduction in death or moderate/severe disability from 50% in the usual care arm to 35% in the intervention (cooled) arm. Using a two-sided 5% significance level and an 80% power, 183 babies per arm are required. Assuming a loss to follow-up rate of around 10%, this comparison requires 204 babies per group, 408 babies in total, to be recruited. In case the adverse outcomes (death and moderate/severe disability) are higher (~65%) in the usual care arm, then this sample size would provide 94% power to detect a 30% relative risk reduction with cooling.

**Fig. 2:**
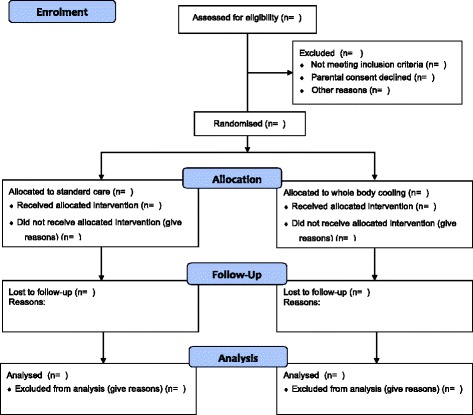
CONSORT *diagram*

Each CRF (pdf) will be send to the HELIX trial manager within 48 h of discharge/death and all data queries will be resolved in real time by the HELIX research nurse/data entry person. The CRF will be signed off for completion and entered into the HELIX Redcap database by a dedicated data entry person at Imperial College London. The HELIX trial manager will then forward the trial data to an independent statistician, after masking the allocation (as X and Y) for analysis. The trial statistician will analyse the data for IDMC meetings as per a predefined proforma (masked as A and B), at six-monthly intervals or after recruitment of every 50 cases whichever is earlier. The temperature data will be reported to the IDMC as adherence to target temperature, rather than actual temperatures to prevent unmasking of the IDMC members. The principal investigators will not have access to the trial data before locking, nor will they have any role in the data analysis. The trial CONSORT diagram is given in Fig. 2.

## Trial organisation

### Independent Data Monitoring Committee (IDMC)

An IDMC will review the study’s progress. The IDMC will be independent of the trial organisers. The IDMC will meet every six months or after recruitment of 50 babies, whichever is earlier.

Meetings of the committee will be arranged periodically, as considered appropriate by the Chair.

The IDMC will inform the Trial Steering Committee (TSC) if in their view:

- there is proof beyond reasonable doubt that the data indicate that any part of the protocol under investigation is either clearly indicated or contra-indicated, either for all infants or for a subgroup of trial participants;
- it is evident that no clear outcome will be obtained;
- safety signal.

The primary endpoint will be assessed 18 months after the intervention has been performed. Given the planned period of recruitment, the primary endpoint will not be able to be assessed until majority of patients have been recruited. Thus, it will not be possible to stop the study early based on the study outcomes.

The membership of IDMC is detailed below:

- Professor Abbot Laptook (Chair, Professor for Neonatology, Brown University, USA)
- Professor Shabbar Jaffar (Faculty of Epidemiology and Population Health, London School of Hygiene and Tropical Medicine, London, UK)
- Professor Niranjan Thomas (Professor of Neonatology, Christian Medical College, Vellore, India)
- Dr Aung Soe (Consultant Neonatologist, Medway Hospital NHS Trust, Kent, UK)

The HELIX trial statistician will provide the trial data for IDMC meetings according to a pre-defined reporting format agreed by the IDMC.

### Trial Steering Committee

The TSC will provide overall supervision of the study of the Sponsor. Its terms of reference are:

1. to monitor and supervise the progress of the HELIX trial towards its interim and overall objectives;
2. to review at regular intervals relevant information from other sources (e.g. related studies);
3. to consider the recommendations of the IDMC.

### Project Management Group

The Project Management Group will oversee all aspects of the day-to-day running of the study and will consist of the investigators and the HELIX trial staff, based at the HELIX Co-ordinating Centre at Madras Medical College, Chennai, India and the Centre for Perinatal Neuroscience, Imperial College London. The Project Management Group will hold a monthly teleconference of all HELIX investigators for the entire duration of the trial to discuss the data quality and recruitment.

All correspondence with the REC will be retained in the Trial Master File/Investigator Site File. Annual reports will be submitted to the REC in accordance with Imperial College London requirements. It is the Chief Investigator’s responsibility to produce the annual reports as required. Each participating site in India will have local research ethics and other regulatory approvals as per the local regulations.

### Consent

The clinical team at each centre will explain the study to the parents, provide study information leaflets (in the appropriate local language – Kannada, Hindi, Tamil, Telugu, Marathi, Bengali, Singhalese or Malayalam) and will seek written informed consent. The entire consenting processes (including explanation of the study to parents) will be video-recorded. The digital video recordings will be securely stored at the local centre and at Imperial College London. The attending physician will regularly meet with parents during the intervention period to ensure that they understand the study procedures, throughout the course of hospital stay.

Each participant’s right to refuse or withdraw from the study without giving reasons will be respected at all times. A withdrawal form will be filled in and authorisation will be obtained for use of the previously collected data.

The site principal investigator will retain the original of each patient’s signed informed consent form. Copies of the information leaflet and consent form will be provided to the parents and kept in the medical records.

### Declaration of Helsinki and Good Clinical Practice

The trial will be performed in accordance with the declaration of Helsinki, the conditions and principles of Good Clinical Practice, the protocol and applicable local regulatory requirements and laws. None of the investigators have any financial or competing interests in the trial results.

### Data protection and patient confidentiality

All investigators and trial site staff involved in this trial must comply with the requirements of the UK Data Protection Act 1998 and local policy (India, Bangladesh and Sri Lanka) with regards to the collection, storage, processing and disclosure of personal information and will uphold the Act’s core principles. The site investigators at each site will ensure that only linked anonymised data are received by the HELIX trial team.

Hard copies of CRFs and consent forms will be stored inside a locked cupboard in a designated HELIX research office at each recruiting centre, under the supervision of the site principal investigator. Only the HELIX research nurse, research doctor, data entry clerk and principal investigator at each site will have access to individual hard copies of the CRFs. Linked anonymised electronic data will be stored in a GCP-compliant secure UK-based server, with daily back-up. The HELIX investigators will have access to the electronic trial data only after the data are locked for the final analysis.

All trial staff must hold evidence of appropriate GCP training or undergo GCP training before undertaking any responsibilities on this trial. This training should be updated every two years or in accordance with the Sponsor’s policy.

### Sponsorship, financial and insurance

The HELIX feasibility trial and the setup of the HELIX trial was funded by the Bill & Melinda Gates Foundation (OPP1069985). The main HELIX trial is funded as a part of a Weston Garfield Chair Endowment Grant (Imperial College London) to Dr Thayyil. The cooling devices (Tecotherm Neo) are provided by Inspiration Health Care, UK, on loan to the recruiting sites.

The trial is sponsored by Imperial College London. Imperial College London will arrange insurance for negligent harm caused because of protocol design and for non-negligent harm arising through participation in the clinical trial.

None of the funders or sponsors have will have any role in the study design, analysis, interpretation or publication of the results.

### Monitoring, audit and inspection

The site principal investigator must make all trial documentation and related records available for the monitoring by the study team and by the Sponsor. All patient data will be handled and treated confidentially.

The study team’s monitoring frequency will be determined by an initial risk assessment performed before the start of the trial. A detailed monitoring plan will be generated detailing the frequency and scope of the monitoring for the trial. Throughout the course of the trial, the risk assessment will be reviewed and the monitoring frequency adjusted as necessary.

### Protocol compliance and breaches of GCP

Prospective, planned deviations or waivers to the protocol are not allowed under the UK regulations on clinical trials and must not be used. For example, it is not acceptable to enrol a participant if they do not meet one or more eligibility criteria (for example, babies with mild encephalopathy or no encephalopathy) or restrictions specified in the trial protocol.

Protocol deviations, non-compliances or breaches are departures from the approved protocol. They can happen at any time, but are not planned. They must be adequately documented on the relevant forms and reported to the chief investigator and sponsor immediately. Deviations from the protocol which are found to occur repeatedly will not be accepted, will require immediate action and could potentially be classified as serious breaches. Any potential/suspected serious breaches of GCP must be reported immediately to the Sponsor without any delay. The SPIRIT checklist is provided as an Additional file 4.

## Discussion

Upon completion, the HELIX trial will provide the most comprehensive data on safety and efficacy of cooling therapy in LMICs. Furthermore, it will provide definitive answers into the interaction of perinatal asphyxia and infection and how this relates to brain injury and neuroprotection in neonatal encephalopathy.

The HELIX trial is unique in several aspects. First, it is a pragmatic trial conducted in the real-life scenario of public sector tertiary neonatal units in LMICs. Cooling therapy will be provided using existing clinical staff in these units and research nurses (if available) will only be involved in collection of the research data. Moreover, HELIX uses exclusive clinical inclusion criteria and examines important clinical outcomes such as death and disability.

The participating centres in HELIX were carefully selected based on the following criteria: burden of encephalopathy (neonatal encephalopathy admission rate > 200 and/or delivery rate > 15,000 per year); availability of tertiary neonatal intensive care including cardiorespiratory support and monitoring; feasibility of long term follow-up; and adequate data quality during the HELIX feasibility trial phase. Advanced intensive care support such as 1:1 nursing care, invasive blood pressure monitoring, nitric oxide and extra-corporeal membrane oxygenator therapy will not be available. Thus, the trial results would be applicable to the vast majority of public sector neonatal units in LMIC. The participating centres have similar guidelines for usual care of encephalopathic babies, although some units admit only in-born babies and others admit only out-born babies. Unlike in high-income countries, withdrawal of life support is not legally permitted in the participating countries.

Substantial inequality in healthcare, both in terms of access and available resources, exists in LMICs. For example, hospitals in the private/cooperate sector (for profit) cater to relatively higher economic strata who can afford to pay for their healthcare and are well resourced. However, these hospitals have lower delivery rates (typically 500–3000 per year) and a very low burden from neonatal encephalopathy. In contrast, publicly funded hospitals (not-for-profit) offer free healthcare to patients from lower economic strata, but are under-resourced and have heavy disease (encephalopathy) burden. The HELIX trial will assess the safety and efficacy of cooling therapy in these socially and economically disadvantaged groups of babies; hence, we will recruit only from publicly funded hospitals (not-for-profit) catering to a low-income population. Thus, the HELIX trial is expected to reduce healthcare inequalities in LMICs.

The HELIX trial results will not be generalisable to settings that lack good neonatal care, as in sub-Saharan Africa. If the HELIX trial demonstrates the safety and efficacy of cooling in middle-income country settings with reasonable intensive care facilities, the next logical step would be to conduct a large pragmatic trial of cooling in sub-Saharan Africa and in rural Indian states, where neonatal intensive care facilities are not available.

Second, given the huge burden of neonatal encephalopathy in LMICs, a modest benefit from cooling therapy on a sub-group of encephalopathic infants, might have a substantial health impact. Hence, several additional state-of-art bacteriological, transcriptomic and advanced neuroimaging investigations will be performed as a part of the research protocol, to examine the interactions of perinatal infection on hypothermic neuro-protection.

We used optimised cross-platform 3 T MR sequences (developed at Imperial College London) so that the data from the three different MR scanner makes (Phillips, Siemens and GE) at the recruiting centres can be pooled together. Before the start of recruitment, we performed extensive harmonisation of the MR scanners and compared the spectroscopy metabolites on the same adult volunteer who travelled to all recruiting different sites. Once completed, HELIX would be the largest trial to use MR spectroscopy biomarkers in neonatal encephalopathy.

Although, none of the advanced investigations (including MRI) would be required in routine clinical practice of cooling therapy, this would provide valuable insights into the underlying disease mechanisms.

### Publications policy

Ownership of the data arising from this trial resides with the trial team. On completion of the trial, the data will be analysed and tabulated and a final study report prepared. Consort guidelines and checklist will be reviewed by the trial steering committee before generating any publications for the trial to ensure they meet the standards required for submission to high-quality peer-reviewed journals, etc. (http://www.consort-statement.org). Sub-studies including the HELIX trial data will be published only after the publication of the main trial results.

A copy of the study results will be also given to the parents of all recruited babies, if they wish. This will be recorded at the time of recruitment and again during follow-up. The Sponsor and funders will have no role in the study management, analysis and interpretation of data, writing of the report or the decision to submit the report for publication.

### Trial progress

The first patient was recruited on 16 August 2015. We have recruited a total of 222 babies at the time of protocol publication (Table 2). The trial is expected to complete recruitment by August 2018.

**Table 2:** Recruitment details

No.	Centre name	Open for recruitment	First case recruited	Total cases recruited	MR scanner	Status
1	Indira Gandhi Institute of Child Health, Bangalore, India	15 Aug 2015	16 Aug 2015	63	3 T Siemens Skyra	Recruiting
2	Institute of Child Health, Madras Medical College, Chennai, India	15 Aug 2015	25 Aug 2015	93	3 T Siemens Skyra	Recruiting
3	Lokmanya Tilak Municipal Medical College, Mumbai, India	31 Aug 2015	5 Sept 2015	40	3 T Phillips Achieva	Recruiting
4	Maulana Azad Medical College, New Delhi, India	On hold^a^	NA	0	3 T Siemens Skyra	Lack of bed space in neonatal unit
5	Calicut Medical College, Kerala, India	Withdrawn^b^	NA	0	1.5 T GE	Withdrawn due to lack of 3 T MRI availability
6	Institute of Obstetrics and Gynaecology, Madras Medical College, Chennai, India	1 Jan 2017	4 Jan 2017	18	3 T Siemens Skyra	Recruiting
7	Bangabandhu Sheikh Mujib Medical University, Dhaka, Bangladesh	7 June 2017	12 July 2017	3	3 T Siemens Skyra	Recruiting
8	University of Kelaniya, Sri Lanka	9 May 2017	24 May 2017	5	3 T Siemens Skyra	Recruiting

^a^Due to substantial increase in hospital deliveries in the hospital since 2016, newborn infants requiring neonatal intensive care unit are having to wait in postnatal wards for several hours before admission. This has prevented research recruitment and, hence, the trial is on hold at this centre

^b^This centre has been withdrawn from recruitment to HELIX due to lack of 3 T MR scanner

## Additional files

Additional file 1: Screening flowchart. (PDF 196 kb)

Additional file 2: Preparation for magnetic resonance imaging. (PDF 303 kb)

Additional file 3: Magnetic resonance (3 Tesla) protocol. (PDF 821 kb)

Additional file 4: SPIRIT checklist. (PDF 77 kb)

## Abbreviations

CRP: C-reactive protein
GMFCS: Gross Motor Function Classification System
IDMC: Independent Data Monitoring Committee
LMIC: Low and middle-income countries
MPRAGE: Magnetization-prepared rapid gradient-echo
MRI: Magnetic resonance imaging
MRS: Magnetic resonance spectroscopy
PRESS: Point-resolved spectroscopy
REC: Research Ethics Committee
SAE: Serious adverse event
SE EPI: Spin-echo echo-planar imaging
STEAM: Stimulated echo acquisition mode
TSC: Trial steering committee
TSE: Turbo spin echo
