# Supplementary material for: Hypothermia for encephalopathy in low and middle-income countries (HELIX): study protocol for a randomised controlled trial
Source: Trials. 2017 Sep 18;18:432. doi: 10.1186/s13063-017-2165-3 (PMC5604260; doi:10.1186/s13063-017-2165-3)
Supplement: Supplementary file 1 — Screening flowchart. (PDF 196 kb) [file 13063_2017_2165_MOESM1_ESM.pdf]

Imperial College London

Centre for Perinatal Neuroscience

# Hypothermia for Encephalopathy in Low and Middle Income Countries (HELIX) Trial

HELIX

## STUDY ENROLLMENT PATHWAY

Baby admitted to the Neonatal Unit with suspected HIE

↓ Yes

Is the equipment available for both cooling and non-cooling groups?

→ No: Patient cannot enroll the study if equipment is being used by other baby (both cooling machine and non-cooling rectal probe must be available)

↓ Yes

Does the baby meet ALL of the inclusion criteria and NONE of the exclusion criteria (see Screening Tool on reverse page)

↓ Yes

Call your Local Helix Trial Consultant to perform NICHD Neurological Examination and obtaining informed consent from Parents.

↓ Yes

According to the NICHD criteria, does this baby have moderate or severe HIE?

↓ Yes

Have the Parents consented for the enrollment of the baby in this trial?

Original CF+PIL filed in medical notes + copies for parents + copies for research folder, + scan saved in local computer

↓ Yes

Once the parents have consented for the baby to take part in the study, you can now randomise the patient (see Randomisation Help Page) and start completing the Case Report Form (CRF).

For any queries, please email helix.study@imperial.ac.uk or call +4420 3313 2473 or +919840653244

# Hypothermia for Encephalopathy in Low and Middle Income Countries (HELIX) Trial

Imperial College London

Centre for Perinatal Neuroscience

HELIX

## SCREENING TOOL

**Inclusion Criteria**

Current age < 6 hours **AND** birth-weight ≥1.8 kg **AND** gestation ≥36 weeks

→ No → Not Eligible

↓ Yes

Any of the following:

- Need for continued resuscitation at 5 minutes after birth
- 5 minute Apgar score <6 (in babies born at hospital) or lack of cry by 5 minutes of age (for babies born at home)

→ No → Not Eligible

↓ Yes

**Exclusion Criteria**

Any of the following:

- Absent heart rate at 10 minutes of age despite adequate resuscitation
- Major life threatening congenital malformation
- Migrant family or parents unable or unlikely to come back at 18 months
- Lack of parental consent

→ Yes → Not Eligible

↓ No

**Inclusion Criteria**

Call Local Helix Consultant for NICHD assessment of severity of HIE and obtaining informed consent

↓ Yes

Evidence of moderate or severe HIE within 6 hours of age (NICHD Criteria) AND parents consented?

→ No → Not Eligible

↓ Yes

Patient is eligible and can be randomised
(Please see Randomisation Instructions)

For any queries, please email helix.study@imperial.ac.uk or call +4420 3313 2473 or +919840653244
