# Supplementary material for: Hypothermia for encephalopathy in low and middle-income countries (HELIX): study protocol for a randomised controlled trial
Source: Trials. 2017 Sep 18;18:432. doi: 10.1186/s13063-017-2165-3 (PMC5604260; doi:10.1186/s13063-017-2165-3)
Supplement: Supplementary file 2 — Preparation for magnetic resonance imaging. (PDF 303 kb) [file 13063_2017_2165_MOESM2_ESM.pdf]

Figure 3. Protocol for MRI scanning of babies on the HELIX Trial

1. All MR scans need to be performed between 4 to 14 days after birth. In exceptional cases where the baby is not clinically stable by this time, the MR scan may be delayed up to 3 to 4 weeks. Please discuss this with the HELIX Chief Investigator on a case by case basis.
2. Local research team should make necessary arrangement to book MRI slot and provide appropriate transport if travelling to another hospital. Patients should be escorted by one doctor and one nurse to and from the hospital where MRI will be performed.
3. The Nurse and doctor accompanying the patient should carry a resuscitation transport bag should the baby need
4. Parents should be informed that we will be expecting to find abnormalities on the brain MR in most babies, prior to the MR scan and explained that the team will discuss the results with them, once the scan has been reported.
5. All infants must have an MRI safety check done **before** the MRI scan. Please arrange this with the radiology team. A list of typical objects which can be unsafe for MRI is given below.
6. A neonatal doctor or an experienced neonatal nurse (trained in newborn life support) should assess the baby to make sure baby if fit for sedation. The baby must not have any respiratory distress (i.e. respiratory rate > 60 min or recession of the intercostal or subcostal areas), and saturations must be over 92% in air before giving sedation for the MR scan. The scan should be postponed if the baby has respiratory distress.
7. Remove any clothes with metal buttons/poppers or any shiny/silver thread and replace them by metal-free clothing. Ensure the baby has had a nappy change before the MR scan.
8. Ear protection must be applied to all infants undergoing MRI, in order to reduce noise exposure.
9. Apply the MR compatible monitor probes (oxygen saturation to fingers/toes of the baby and temperature probe to the chest/axilla of the baby). Use a thin linen or sheet to wrap around the cables and prevent contact with the infant's skin and aim to keep all cables straight.
10. Give a breast/NG/bottle feed approximately 60 minutes before the MR scan.
11. Give sedation: Oral / NGT / PR Chloral Hydrate, maximum dose 50mg/kg, only one dose should be given. This should be given at least 30 minutes before the MRI scan and ideally 30 min after the feed. If using PR Chloral, make sure the suppository is not expelled by bowel action.
12. Monitor the baby in a quiet dark place for 30 minutes before the MR scan, so that baby can settle asleep and avoid disturbing as much as possible.
13. Just before the MR scan place the baby on MedVac® mattress.
14. Gently transfer the baby into the MR room and place inside the head coil of the scanner.
15. A neonatal doctor or an experienced neonatal nurse (trained in newborn life support) should monitor the oxygen saturation, heart rate and temperature of the baby continuously during the MR scan and enter this into the monitoring form provided. This form should be filed in the patient's notes after the scan. Essential equipment for neonatal resuscitation including an ambu bag, different sized masks, intubation and suction equipment, emergency neonatal drugs, should be available in the MR suite. In the event of an emergency, local MR team should place a crash call according to local policy to ensure the fastest possible response. There should be facilities to summon immediate help from the hospital resuscitation team in case of a respiratory or cardiac arrest in the MR room,
16. All babies given sedation must be observed for a minimum of two hours after the MR scan. Prior to discharge home, the baby must be awake, have taken a good feed, and must have had an examination by the doctor or neonatal nurse.
17. The MR scan should be reported by the local radiologist, and the report fed back to the parents by the local site investigator/clinical team, and documented in the medical notes.

Ferromagnetic checks before MRI Scan.

All subjects MUST have a metal check performed **before** entering the MRI scanner. The table below is not an exhaustive list of all possible items but include common items you will need to check for safety. Please refer to local policy to check and confirm all items to be MR compatible.

Any object is assumed to NOT be compatible with the MRI scanner until proven otherwise. To be considered safe for scanning the exact specifications of any internal metallic object (PDA clip, intra-ventricular reservoir, etc) must be known and discussed with the radiographer before a child enters the scanner.

**Typical objects that need to be checked for MR safety**
Vascular Lines:<br>Arterial lines<br>Umbilical lines<br>Long lines
Fixation devices for lines, e.g. splints
PDA clips
Surgical implants/any history of surgical procedures
Scalp needles
Electronic name tags
Name tags with metal closures
Clothes with metal poppers
Ward pulse oximetry
Ward ECG leads
Ward temperature probes
Religious artefacts
Endotracheal tube holders
**Other important checks**
Baby had cares done (dry nappy)
Hearing protection applied

Applying Ear Protection before the MRI Scan

The MRI scan typically generates noise levels above 100dB. By applying the following protection, we expect to reduce the noise levels substantially. Ear protection must be applied to all infants undergoing MRI.

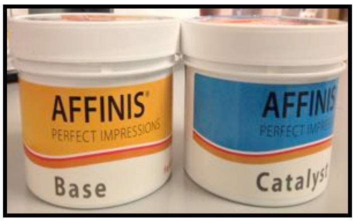

1. Mix equal parts of both components of the dental putty until you form an homogenised paste

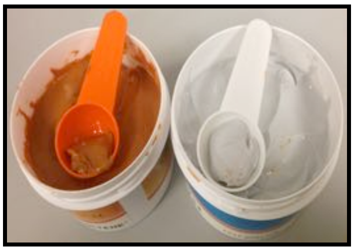

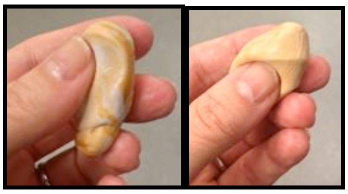

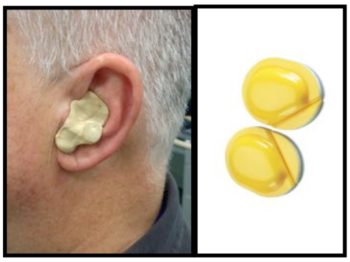

2. Apply the putty to cover the ear while soft (putty will start to harden).

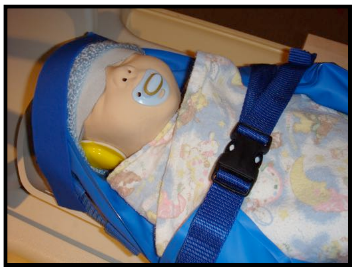

3. Apply ear muffs. Support with hat and/or velcro strap. Phototherapy eyeshades can fit both purposes of helping secure the earmuffs as well as helping baby to close the eyes and sleep. Baby is now ready to go into the MedVac®

4. After the scan, the earmuffs and putty can be easily removed without leaving any residue.

Applying MedVac® Infant immobiliser

MedVac® is an air-tight vacuum-chambered splint that provides effective immobilisation during the scan. The mattress is wrapped around the baby using velcro straps and the air inside the mattress is evacuated, so that it forms a firm mould around the baby.

- MedVac® can be set up outside the MR room, after a thorough metal check and appropriate positioning of the baby.
- All other checks, including ear protection etc. should be completed before placing the infant on the mattress.

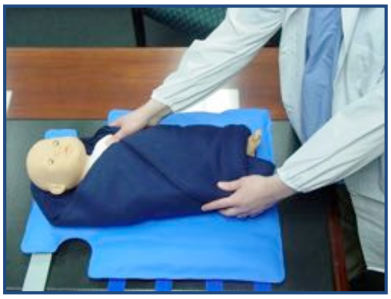

1. Wrap the baby in a thin sheet within the MedVac®. Leave any extremity used for saturation monitoring uncovered, enabling easy access to this.

2. Make sure the valve on the mattress is tightly closed. Place the infant on the mattress. Use the straps provided to loosely wrap it around the infant's body (and head if needed).

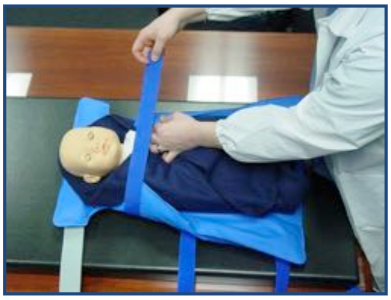

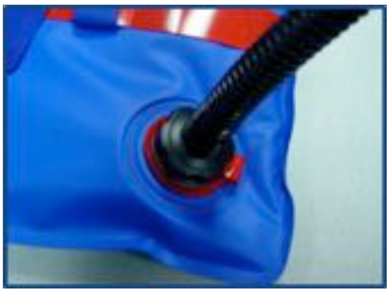

3. Evacuate the air within the mattress through the valve using either the wall mounted suction or hand pump provided.

4. Detach the hose and the baby is ready to be taken into the scanner room.

5. To re-inflate, simply open the valve.

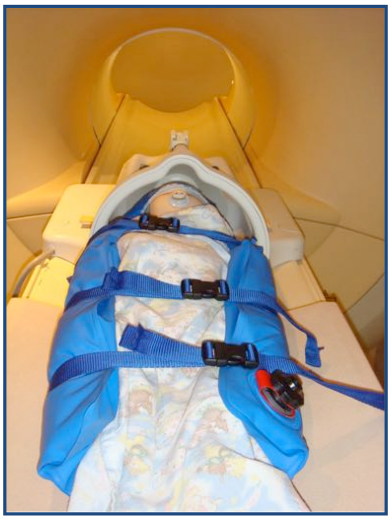

6. With the infant in the head coil, foam padding should be placed between the head and MedVac® for additional hearing protection.

For further instructions, please contact the Chief Investigator or visit the MedVac® manufacturer's website at: http://cfimedical.com/medvac/

Monitoring Form (for all babies undergoing MRI as part of Helix Trial)

Patient Name

DOB

Hospital Number

Date of Scan: ___/___/_____

Start Time:___:___

End Time:___/___

Sedation Details:

Drug used:_____________________

Dose:____________ Time:____:____

**Location**	Please circle as appropriate: **D** Departing Neonatal Unit – **M** In MRI – **L** Leaving MRI – **A** Arrival in Neonatal Unit																			
	DMLA	DMLA	DMLA	DMLA	DMLA	DMLA	DMLA	DMLA	DMLA	DMLA	DMLA	DMLA	DMLA	DMLA	DMLA	DMLA	DMLA	DMLA	DMLA	DMLA
**Time**																				
**Axila Temp.**																				
**HR**																				
**SatO_2**																				
**RR/Vent.**																				
**Any change**																				
**Initials**																				

Notes:
