# Supplementary figures and images for: Hypothermia for encephalopathy in low and middle-income countries (HELIX): study protocol for a randomised controlled trial
Source: Trials. 2017 Sep 18;18:432. doi: 10.1186/s13063-017-2165-3 (PMC5604260; doi:10.1186/s13063-017-2165-3)

Figure 1: Outline CONSORT diagram:

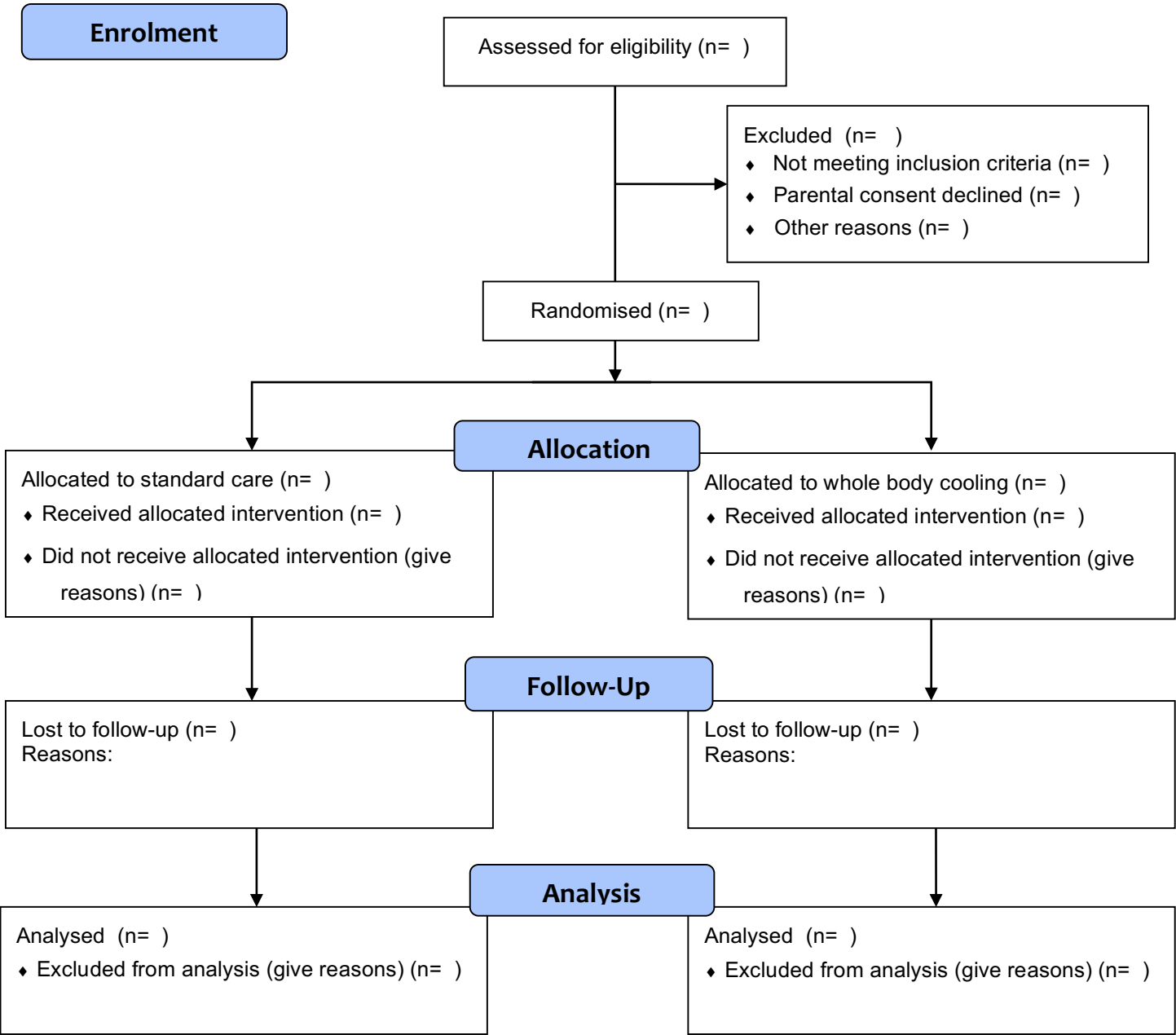

Supplement: Supplementary file 3 — Magnetic resonance (3 Tesla) protocol. (PDF 821 kb) [file 13063_2017_2165_MOESM3_ESM.pdf]
